# Supplementary material for: Recurrent neural networks as neuro-computational models of human speech recognition
Source: PLoS Comput Biol. 2025 Jul 28;21(7):e1013244. doi: 10.1371/journal.pcbi.1013244 (PMC12331064; doi:10.1371/journal.pcbi.1013244)
Supplement: Attachment — Submitted filename: R1 - response to reviews.pdf [file pcbi.1013244.s002.pdf]

Dear Editors and Reviewers,

We thank you for the positive and insightful comments. We have revised the article to address your concerns and suggestions. We believe this has greatly improved clarity and added interesting additional results and points of discussion.

One point, raised by both reviews is that our deep models had more predictors for neural activity than the flat models (R1, #2 and R2, #5). To address this, we used K-mean clustering to create alternative unit groupings for all models. K-means based groupings increased predictive power over the grouping based on hierarchical layers in our original manuscript. These new results favored slightly different architectures, which led us to redo much of the later analysis with newly trained models. Interestingly, this did not change most of the major conclusions, speaking to the robustness of these findings. However, given that unit grouping by K-means clustering led to higher predictive power than grouping by hierarchical layer, we felt that this undermined the motivation for the anatomical analysis by layer, which we have thus removed (former Figure 4). While we feel that the analysis of hierarchical unit groupings deserves attention, a new approach is needed, which is beyond the scope of the time given for these revisions.

We also have to defer some very interesting suggestions made by reviewers for future work, where these issues can be appropriately addressed (e.g., comparison to automatic speech recognition of continuous speech, and including lexical frequency in simulations).

Please find a detailed point-by-point response below.

Sincerely,

Christian Brodbeck, Thomas Hannagan & James S. Magnuson

Reviewer #1

In this paper, Brodbeck et al. explore how RNNs, particularly LSTMs, can be used to model and understand the neural processes involved in human speech recognition. They trained LSTM RNNs to recognize speech from auditory spectrograms and tested whether these networks' internal dynamics can predict human neural responses to the same stimuli. They found that the RNNs' predictions of neural responses improved when the network architectures were modified to include cognitive principles like phonetic competition, suggesting a more human-like processing of speech. Furthermore, they demonstrated that RNNs can replicate some aspects of the hierarchical nature of neural processing in the brain, with different layers of the network modeling different levels of brain response to speech.

Overall, the paper is well-written and logically structured. I do have several comments and suggestions that could potentially strengthen the manuscript:

1. Temporal Dynamics and Phonetic Competition

The manuscript claims that modifications in the LSTM loss function enhance the model's capability to capture phonetic competition, leading to better neural response predictions (as shown in Fig 3). However, it remains unclear if the additional variance explained by the new model with the modified loss function directly results from phonetic competition. The paper does not empirically demonstrate the existence of phonetic/lexical competition in the MEG activity. It is also not clear the emerged competition effect in the LSTM hidden units can be correlated to these MEG components. Without isolating the effect of phonetic competition from other factors, it might be premature to attribute improvements in prediction accuracy solely to this aspect.

We do not mean to imply that phonetic competition is *directly* encoded in LSTM (or brain) activity, nor that the amount of lexical competition is a mediating variable between LSTM and brain activity. Rather, our observation is that the information processing dynamics in the models with altered competition correspond more closely to human brain dynamics. For example, it is conceivable that a model with original loss function performs more work early during a word when it is attempting to isolate the target quickly, whereas a model with modified loss function performs more work later on when it is still distinguishing between onset competitors. We have rephrased the relevant sections of the Results and Discussion to make this clearer.

> 2. Model Complexity and Prediction Accuracy
>
> Figure 2G can also be interpreted as increasing the number of features in the mTRF model enhances brain prediction accuracy. This observation raises a question whether clustering the units into more clusters based on their activation profiles would lead to better neural prediction as well, since the grand averaging across all units may attenuate the between-unit variance. The extreme case would be to use activation of each unit as a distinct predictor, instead of averaging across units.

Thank you for pointing out this confounding issue. To follow up on this suggestion we used K-mean clustering to create alternative unit groupings. K-means based groupings indeed increased predictive power over the grouping based on hierarchical layers. The new analysis employing K-means clustering did not change any of the major conclusions regarding model architecture. However, given that unit grouping by K-means clustering led to higher predictive power than grouping by hierarchical layer, we felt that this undermined the motivation for the anatomical analysis by layer (former Figure 4), which we have thus removed.

> Another concern I have is that the heavily unbalanced number of predictors. The baseline have 256 features, while the RNN predictors only have 2 features. Would increasing the RNN predictors change the results?

We apologize for the lack of clarity – the baseline model included 17 features (8 band spectrograms, 8 band onset spectrogram, and word onsets). We added a sentence to the Methods section to make this unambiguous. Regarding the latter point concerning increasing the RNN predictors, we now show that increasing the number of RNN predictors using K-means clustering does indeed improve predictive power.

> 3. Extension to Longer Linguistic Contexts
>
> The current focus of this manuscript is primarily on single-word perception, which relies heavily on model training specifics (as noted in Fig 2C). Given RNNs' capability to capture long-term dynamics, it would be valuable to see how this model performs with longer linguistic contexts, such as sentences. For instance, could pre-training the model on a task like automatic speech recognition or using pre-trained models like Deep Speech 2 influence the emergence of dynamics similar to those depicted in Fig 3I?

We completely agree, and think this is a logical next step to pursue. However, this is a non-trivial extension of the paradigm we report here for a variety of reasons (starting with technical issues, such as extending localist target space to a vocabulary appropriate for automatic speech recognition). We thus have to leave this for future work.

Minor Points:

1. The labeling of panels ABCDE in Figure 1 is somewhat confusing. Consider reordering these labels to reflect a top-to-bottom, left-to-right progression to enhance clarity.

We have reorganized the labels on Figure 1 to follow left-to-right progression.

2. In Figure 1, the feature is described as the summation over the absolute values of hidden unit activations, yet the absolute term appears to be omitted in the graphical representation. Clarifying this in the figure could prevent potential misunderstandings.

We have added the absolute operation to the graphical representation.

3. Is there any regularization included in the mTRF model? Given the dimensionality of the regression problem, it may be worth considering some sort of regularization, such as L2. I am also curious why the authors used coordinate descent as the solver for the regression.

The TRF estimation includes regularization. We have made this more explicit, and also added a supporting reference.

Reviewer #2

I'm pleased to provide a review of this very interesting paper. The paper is well written, clearly organised and addresses a topical and important question in advancing our understanding of the neural and computational mechanisms that support perception and identification of spoken words by human listeners. This is a domain in which engineering systems have achieved considerable success - achieving near-human levels of accuracy even in challenging listening situations - by applying modern neural network methods. However, human-level performance need not imply human-like mechanisms and this paper therefore explores the architectural, representational and computational assumptions required for a recurrent neural network system to show human-like behaviour and neural activity.

The paper achieves some success in explaining both behavioural and neural data and thus makes a very useful contribution to this area of computational biology. However I had some comments on the relationship between the computational simulations and behavioural and neural outcomes which I think would be best addressed by revisions to the manuscript and further consideration by the authors. I'll discuss these two areas in turn, before adding some minor comments about specific points of presentation.

(A) Relation to behavioural data

(1) The first indicator of human-level behaviour are low word-error rates (WER). These are typically very low for skilled listeners (5-10% or fewer, depending on listening situations, stimulus materials and task/scoring criteria). Established DNN models already achieve similarly low error rates (e.g. Kell et al, 2018 Neuron). Interestingly, the model explored in the present work did not previously achieve such low error rates (this is the EarShot model from Magnuson et al, 2020, for which WER was around 30% - though this depends on the test and generalisation conditions that are considered most comparable). I think it would be useful for the authors to make this comparison with the previous version of Earshot more explicit. Is their model substantially different from and better performing than previous versions of EarShot? Which of the previously observed WER are most comparable to this new model? How does the change from the purely-synthetic speech used previously to the mixed natural and synthetic speech on which this model is trained impact WER and other measures? I think the authors should explain why this model is (seemingly) so much more accurate.

We agree that this would provide valuable context, and have added this comparison to the relevant Results section (*Sparse output spaces improve word recognition*). In brief, the performance of the model that is most comparable in our new data is actually quite similar to the model in Magnuson et al..

(2) One of the most interesting aspects of the original Earshot model, that is shared by this updated model, is that - while working with neurally-plausible temporal processing mechanisms (i.e. sequential audio input, and recurrent connections) - it accurately simulates the time-course of word identification, capturing well-established behavioural findings concerning the time-course of recognition of onset-aligned "cohort" competitor words (such as beatle and beaker). In the previous EarShot paper (Magnuson, 2020) this property was demonstrated in the main simulation, but changes to the input representation prevented this being observed (Supplementary Figure S1.3 using MFCC input). The present paper reports similarly variable findings in Figure 3D-F. However, it's not clear that these figures sufficiently justify the authors statement that "activation of the target was delayed reflecting the word-initial acoustic ambiguity".

It seems that the model is recognising target words at around 300ms, whereas the time-course of the input for words like conserve/concerned (in Figure 2H) would suggest that this is still rather early given the uniqueness points of some of the spoken words. While the depiction of the time-courses in Figure 2I are more persuasive, and the figure style used is impressively compact, I think that a more direct assessment and clearer depiction of the relationship between stimulus timing, stimulus uniqueness points and model recognition points would be helpful. Is there statistical evidence that recognition is time-locked to word uniqueness point rather than word offset in these simulations? Will these models capture differential effects of uniqueness point on recognition of words that are higher vs lower frequency members of their cohorts (suggested, for instance, by Dahan et al, 2001 or Gaskell & Marslen-Wilson, 2002)? My understanding of the literature is that these forms of behaviour are well-approximated by models that activate words in proportion to their conditional probability given the speech input heard thus far (in line with Norris & McQueen, 2008). Does the current model achieve this?

We have added data showing that, in contrast to the model responses to human tokens that lack cohort competition, responses to synthetic talkers exhibit cohort competition as expected (Figure-4-C). This is consistent with the result of realistic cohort competition in Magnuson et al. (2020), which used only synthetic talkers. The lack of cohort activation thus seems to be due to some feature of the human talkers. We have considered a possible explanation for this in the *Discussion* under *Loss function for word recognition*.

We cannot directly assess effects of word frequency as training tokens are sampled from a uniform distribution. However, we agree that lexical frequency is an important target for future work.

(3) In their later simulations the authors separate two aspects of the typically-used binary, cross-entropy error term by adding an additional parameter ("c") which reduces the penalty faced by the model for activating competitor words early on in the identification of specific spoken words. This achieves the desired outcome by (I think) relaxing the assumption that the model is performing multi-class classification (i.e. 1/N words are present at a time), and making the model perform something more similar to

multi-label classification (i.e. several words may be simultaneously present and hence co-activated). This seems like the wrong assumption - the speech signal contains only one word at a time - but perhaps I've made a mistake in my interpretation here (I should note that one of my colleagues who co-reviewed didn't agree with my interpretation). Nonetheless, I had some concerns with the presentation and interpretation of this result, perhaps due to some very compact graphs and explanation, and perhaps also due to some problems with the behavioural and computational interpretation. Specifically:

Binary cross-entropy is already consistent with multi-label classification, because it independently assesses the difference between label and prediction for each output element (see equations that we have added to *A modified loss function for human-like lexical competition* under *Results*). However, the intuitive 1/N constraint comes in through the fact that the loss can be minimized by predicting the probability of a given label (as probabilities for words sum to 1). We have added corresponding discussion to *Results*, *A modified loss function for human-like lexical competition* and *Discussion*, *Loss function for word recognition.*

- in the main text and in figure 3I there's inconsistency about whether c is used as a divisor, or whether some error terms are multiplied by 1/c. Of course, these are mathematically equivalent, but think being consistent in their presentation (e.g. showing c=16, or c=1/16 in both the text, figure and equations) would be more transparent for readers. I would also ask the authors to report the precise form of the cross-entropy error equation that they are using in the simulations including the "c" parameter and to consider how this relates to forms of the Cross-Entropy Error term used for multi-class and multi-label classification.

We have update the relevant Figure labels. In the section *A modified loss function for human-like lexical competition*, we have also added the full error equation and extended the discussion of the loss function, including the relationship to multi-label classification.

- I would also ask the authors to reflect on why this modified cross-entropy term was not necessary in order for the earlier EarShot model in the Magnuson 2020 paper to show appropriate cohort competition effects. I couldn't find a specific description of the error term used in this earlier paper, but I assume that this is cross-entropy error given the sparse binary output vectors used. Indeed, I know from personal experience that other (highly simplified) recurrent network models explored in the 1990s (definitely Davis et al, 1997, but perhaps also Norris, 1990; or Content & Sternon, 1994) used cross-entropy error with localist output units without this modification and still showed cohort competition effects (see Figure 2 and 3 of Davis et al, 1997 for example).

Thank you for bringing up this question. We have added responses to synthetic stimuli to the lexical activation plots, showing that there is indeed competition in response to those stimuli. We have added some speculation on the difference between human and synthetic speakers to the *Discussion* under *Loss function for word recognition*.

- It's not clear whether their model with appropriately tuned values of the "c" parameter (Figure 3I) still achieves probabilistic activation of target and competitor words (as explained in Davis et al, 1997, at each time point during a word, localist output units approximate the conditional probability of each word being present given the current input). I note that in Figure 3I the summed activation of target and competitor words clearly exceeds 1. I therefore think these output values no longer approximate conditional probabilities (which should, by definition sum to 1). Does this matter? Is it still the case that the other desirable properties of probabilistic activation (discussed in Norris & McQueen, 2008) still apply for this model? For example, will the model still show preferential activation of higher frequency words for cohort competitors that are mismatched in frequency (see (2) above)?

This is an (intended) consequence of the modified loss function. While reading word activation as probabilities may have advantages from a machine learning perspective, we are not aware of any evidence that activation in neural representations of lexical representations ought to have a constant sum. We have included this consideration in the extended motivation for the modified loss function under *A modified loss function for human-like lexical competition*, and in the *Discussion* under *Loss function for word recognition*.

We can still derive probabilities by renormalizing the resulting distribution (as shown in Figure 4-B). Although we were not able to test this prediction, we would expect that lexical frequency would still influence activation strengths, because the loss function is scaled equally for all non-targets. Our results do show that cohort competition reflects the number of shared onset phonemes, i.e., the modified loss function at moderate levels leads to sensible lexical hypotheses, and not simply to indiscriminate lexical activations.

- I also wonder whether a training protocol that changes the way in which error terms are computed at specific points in a word could plausibly be implemented in the brain? Does this require that listeners anticipate when each word ends in advance? Or is there some other training protocol - e.g. back-propagation of error being applied only at word offset - that would help here? I note that there's a red box drawn at the end of each word's "training signal" in panel C of Figure 1 which could suggest this. A more detailed explanation of the time-course of the training procedure, and exploring whether model outcomes are sensitive to whether the pre-offset parameter is 200ms (rather than, say, 100 or 300ms), might be helpful in clarifying what's going on here.

How the training procedure relates to neural learning is a very interesting question. We do not consider our work to be developmentally realistic in that respect, but this is definitely an important question for future work. We have added a corresponding *Discussion* subsection at *Loss function for word recognition*. We have also clarified the target signal in the Figure 1 legend.

(B) Relation to neural data

The most novel and important contribution of the present paper to the literature is that the authors use several different variants of their model - varying output representations, and model depth while keeping the total number of parameters relatively constant. They then explore whether measures of aggregate neural activity and change in neural activity in these different models predicts MEG data collected when participants recognise the same spoken words presented to the model. The approach used builds on previously published MEG data which was analysed with TRF-encoding methods by Gaston and colleagues (2023). They show that for (essentially) all of the simulations reported their neural network derived predictors substantially improve the amount of variance explained in MEG data compared to an acoustic "baseline" model which includes a spectrogram, onset-spectrograms, and word onset events. This success is impressive and builds on previously published demonstrations that measures of the internal operation of neural network models can help explain fMRI observations (Kell et al, 2018, Neuron). I had a few questions about the reason(s) for the success of their model in predicting neural data. I think some consideration of this issue would help ensure that the present work can be readily interpreted by readers who do not have access to the advanced simulation methods used in the present paper.

(4) What is the relative contribution of summed activity and change in activity? Can the authors report the variance explained by each of these different measures? I'm personally very interested in the idea that change in activity - perhaps related to moment-by-moment prediction error or surprisal - is especially predictive of neural responses. By separating the explanatory value of these two measures they might offer relevant evidence to assess predictive processing theories. I would also ask if summed and change in activity relate in the same way to neural data - for instance do both these model outcomes predict a positive impact on MEG responses? Or is a one a positive and the other a negative predictor?

The two predictor types contributed to similar extents. We have added the % variance attributable to each predictor type for the final mode to the *Methods* section where we describe the predictors: “In the final model (3*256, c=256, K=32), 27.0% of the variance predicted by the RNN could be uniquely attributed to the magnitude predictors ($t(17)=5.91$, $p<.001$), and 29.8% to the change predictors ($t(17)=7.30$, $p<.001$).”

The question regarding the sign of the regressors is complex. First, typical TRFs often exhibit both positive and negative peaks (see e.g., Brodbeck et al., 2020). Second, because our dependent measure is (signed) source current, it is not actually clear whether a positive or negative sign in the TRF increases or decreases predicted currents overall. Because of this

added complexity we did not add any additional analysis, however, we would be happy to share all relevant data and code to allow others to pursue such an analysis.

> (5) Another impressive aspect of the present work is their exploration of the impact of changes to network architecture on the neural variance explained. They persuasively show that deeper networks provide additional explanation of observed neural data, particularly when used with semantically structured, Glove output vectors. However, it's not sufficiently clear whether the improvement observed is due to deeper networks being more human-like, or whether this is due to their being additional parameters in the linear regression that relates network activity and neural data. For example, in Figure 2G is the marked improvement in variance explained due to separating two forms of hidden and output units? Or due to their being more free parameters (4 rather than 2) when using TRF regression to predict neural data? The same question could be asked when considering models at different depths (in Figure 3C)? Do their neural predictions from deeper models involve using separate predictors from each multiple layers? Or is it the case that it's only activity in the last layer of the deeper networks which is used to predict neural data?

Thank you for pointing this out. This concern corresponds to Reviewer #1's Point 2 – please see response to that point.

> (6) I will also admit that I'm unfamiliar with the separation - introduced in Figure 2E and other simulations - of local, recurrently connected hidden units, and other units that are connected to output units. Is this a common manipulation introduced in LSTM networks? How does this differ - in computational complexity and efficacy - from simply adding a second layer of hidden units? Is this difference between locally-connected and output-connected hidden units preserved in their later simulations? The presence of two graphs for "6-local" and "6-out" in Figure 4A suggests that this is the case. It would be helpful - in Figure 3A, for example - to show more details of the way in which all the deeper networks are organised and to discuss the difference between splitting a single set of hidden units, and adding an additional hidden unit later in more detail.

Our re-analysis with K-means clustering suggested that fully connected flat models with K=2 were generally more predictive of brain activity than the models distinguishing locally-connected and output units. This undermined our primary motivation for this architectural feature. Also considering the complexity of this manipulation (as evidenced by the open questions in this comment) we decided to drop it from the revised manuscript and only work with fully connected output layers.

> (7) In understanding why it is that later layers of deeper networks explain more variance in neural data it would be helpful if the authors could depict and explain the general properties of the aggregate activity and change in activity observed in the model during the timecourse of a spoken word. As depicted in Figure 4A, it seems that early layers that show a time-course correlated with amplitude envelope - is it possible to quantify

> this more directly? For later layers in these deep networks, units might show a less acoustically-responsive profile, but perhaps they could confirm this statistically (using correlational or RSA-type analyses).
>
> Even with this explanation, however, it's still not clear why and how the temporal profile of later/deeper processing layers relate to position within a word. Does activity increase over time within each word? Or does activity relate to information theoretic or psycholinguistic predictor variables - e.g. word length, uniqueness point, cohort entropy, phonemic surprisal, etc. If it were possible to explain the operation of the neural network in these terms it would help open up this black box and to explain why these recurrent networks are such good linking hypotheses in explaining neural data.

Given that we had to drop the anatomical analysis from the current manuscript, this analysis becomes impossible. While we would be very interested in how model activity is related to other psycholinguistic variables, such an exploration would be open-ended and would further delay this manuscript.

However, as far as we can tell, obvious psycholinguistic variables are unlikely to mediate the predictive power of the RNN for neural data, because the predictive power of phoneme surprisal for the same MEG data was far below that of the RNN, and cohort entropy and the uniqueness point did not significantly improve brain predictions (Gaston et al., 2023). We have added a brief section at the end of the *Results* highlighting the difference in explanatory power, *The RNN explains more variance than phoneme surprisal*.

> (8) This challenge of relating different neural layers to specific neural sources is most clearly expressed in Figure 4D in which the authors explore whether different network layers predict activity in different neural regions. While the authors suggest that lower network layers predictive activity close to auditory cortex and higher network layers predict activity in other areas it seems just as accurate to say that the difference is more in the strength of correlation with neural activity as in the spatial location of predicted activity. In order to interpret these results as showing hierarchical correspondence, the authors need to confirm that there's a cross-over interaction such that early auditory areas are better explained by lower layers of the neural network and that higher lexical processing regions are better explained by higher layers of the neural network. The presentation of thresholded statistical maps can be persuasive to readers but does not suffice to confirm regional specialisation. What is required is the sort of analysis shown (for fMRI data, and using more conventional statistical modelling methods) in Figure 7 of de Heer et al (2017, Journal of Neuroscience). That is, confirming differential spatial tuning of relevant neural responses. In the absence of these analyses, a more cautious presentation is required.

Thank you for this note of caution, which we will take into account in future work.

Other/minor comments:

- Page 3-4 - the authors are right to talk about how many "transformer" models (e.g. BERT) make implausible assumptions about the nature of the temporal processing that is applied to speech or language stimuli (since left and right context must operate differently). However, they need to take care in distinguishing between different models since many current language models do not work backwards in time. My understanding is that all "generative" models necessary operate using "causal" mechanisms (ie. only forward in time). I've seen a distinction made between causal and acausal models and they might find these terms helpful here.

Thank you for pointing out this ambiguity. We have clarified that we are referring to Transformers for speech recognition, not generative language models. However, we believe the argument could still be extended to most language models: While it is true that generative language models predict one word at a time, each of those predictions is generated by providing the whole left context as input.

- Page 4, para 4 - they describe their model input as a "continuous spectrogram", but I think they're using discrete words (without coarticulation), and present single spectrogram slices at a time. It's not clear that "continuous" is a useful term here.

We have removed “continuous” and added a sentence to explain our originally intended meaning (that LSTM states were not reset between words).

- Figure 2A / B / C - I found the depiction of these networks confusing. There's a level of "input" units that are shown as a graph. But, then there's a "dense" layer *before* the output graph. Does that mean the network has two layers of hidden units? I think not, but can't be sure. Furthermore, the labelling of the output as "dense" contrasts with the description of "Random Sparse Vectors" (RSV). EarShot similarly labels these outputs as "sparse". Is one of these labels in error?

Thank you for pointing out this ambiguity. We have removed the “dense” label, which was intended to characterize the dense connectivity between LSTM and output.

- Figure 2E - dividing the recurrent layer into Hidden only and Hidden Unit + Output is seemingly not the same as having two HU layers in Figure 3. But why and how is the connection patterns and number of parameters different from the later simulations with two layers of hidden units?

We have removed these models in the revised manuscript.

- Figure 3 - when showing networks with "multiple hidden unit layers" (Figure 3A) I think it would be helpful to depict these in the same way as the depictions in Figure 2. For their best performing "3 layer" model in Figure 3D is this a model with "3 layers" (input / HU / output) or "3 HU layers" (input / HU1 / HU2 / HU3 / output)?

The depictions are now consistent (apart from Figure 3-A omitting input and output).

- Figure 3D / F / I - In plotting the output of these model I think that they're showing output activation for single, localist units. Is tht correct? Or is this a cosine similarity - like shown in similar Earshot simulations?

Yes that is correct, the plots show model output values in the localist space. We have clarified this in the figure legend.

- Figure 4B/C - it's helpful to show similarity between different layers. It's striking to me that there's not always maximum similarity along the diagonal (that is, adjacent levels are not always maximally similar). Does this imply that there's processes that skip intermediate levels? Or does it suggest that (as argued by Kornblith et al), that simple correlations between layers is not necessarily a good measure of representational similarity? Also, it would be helpful to show similarity to input and output representations in these figures.

We have removed the corresponding analysis in the revised manuscript due to the superiority of K-means clustering over predictors based on hierarchy.

- Figure 4D - This figure aims to set out correspondance between early layers of a network, and the the spatial organisation of the auditory/speech/language processing hierarchy. I would be interested to know whether earlier and later TRF effects (ie. the temporal organisation of neural responses) similarly corresponds between their neural networks and brain responses.

We have removed the corresponding analysis in the revised manuscript due to the superiority of K-means clustering over predictors based on hierarchy.

Signed - Matt Davis, Cambridge, uK
