# Supplementary material for: Recurrent neural networks as neuro-computational models of human speech recognition
Source: PLoS Comput Biol. 2025 Jul 28;21(7):e1013244. doi: 10.1371/journal.pcbi.1013244 (PMC12331064; doi:10.1371/journal.pcbi.1013244)
Supplement: Attachment — Submitted filename: R2 response to reviews.pdf [file pcbi.1013244.s003.pdf]

Dear Editors and Reviewers,

We sincerely thank you for your time and effort on our manuscript. We have revised the manuscript based on your feedback; we hope you will agree it is substantially improved and ready for publication. Please find a detailed point-by-point response below.

Sincerely,

Christian Brodbeck, Thomas Hannagan & James S. Magnuson

Reviewer #1

> I appreciate the authors' efforts in revising the manuscript and commend them for addressing most of my previous concerns. I have only one more suggestion for further improvement:
>
> The authors claim a progression of neural similarity with increasing layer depth. Is there a direct evaluation of the embedding space of the RNN layers? While Fig. 3B illustrates the WER across layers, which partially addresses this, it would be insightful to examine whether deeper layers capture more phoneme- or word-level structures compared to acoustic-level representations. To strengthen the analysis, the authors could consider performing the analysis in Fig 2F/2G across different depth layers. Or the authors may also consider a more direct evaluation similar to the approaches proposed by Pasad et al. (ASRU 2021) or Belinkov & Glass (NeurIPS 2017).

As suggested by the reviewer, we have applied the analysis of the learned output space, previously shown in Fig 2F, across models with different layer depth (Figure 3-E) and to models with the modified reward function (Figure 4-L). Interestingly, this additional analysis revealed that, while phonetic structure is somewhat reduced in deeper models, it largely persists and is enhanced by the modified loss function (as may be expected in a model that exhibits phonetic competition).

References:

Pasad, A., Chou, J. C., & Livescu, K. (2021). Layer-wise analysis of a self-supervised speech representation model. 2021 IEEE Automatic Speech Recognition and Understanding Workshop (ASRU), pp. 914-921. IEEE.

Belinkov, Y., & Glass, J. (2017). Analyzing hidden representations in end-to-end automatic speech recognition systems. Advances in Neural Information Processing Systems, 30.

Reviewer #2

Thanks for the opportunity to review this revised manuscript. As before, I am interested by the work and mostly satisfied with the revised manuscript that the authors have produced, and by their responses to my comments and suggestions.

I had only one substantive point in the section reporting "a modified loss for function for human like lexical competition" which I think the authors should do a bit more work to address. I also had a very few minor corrections and typos.

Presentation of lexical competition findings:

One strength of this manuscript is the way in which the authors take seriously the functional requirement that words be identified rapidly and in time with the speech input. This was not a feature of previous models (e.g. Kell et al, 2018, Neuron) which were otherwise modelled on human performance. It is very interesting to me that - by default - the model does not seem to simulate the appropriate time course for human speaker tokens, and only approximates this time-course for unseen synthetic speaker tokens. The correct, human-like timecourse becomes more apparent when the network reward function is modified to reduce the functional pressure against selecting a single word, and permitting parallel activation of multiple word candidates, this reduces word error rate and improves neural prediction.

However, I still feel that I don't understand a few details of the time-course of the activation profile for the original simulations, and for those with the modified reward function shown in Figure 4. Specifically:

- how is the original model able to identify target words so early - well before their phonemic uniqueness point? It is shown in the paper that target activation is delayed when human speaker tokens were untrained (Figure 4D). But, this still seems like very early activation compared to the more appropriate time-courses shown in Figure 4F (e.g. with C=256, or C=4096). I am troubled that it seems as if the model can predict which word is being heard before any disambiguating information has been heard! I would like the authors to keep this concern in mind in presenting their results. Are there additional details of the model, the speech tokens, or the timecourse of activity that are relevant here? If - as the authors state - this effect is not due to "overleaning of specific tokens" then what could be the cause of this?

We thank you for insisting on this point. Further investigation has revealed that our training procedure led to contamination of the training set (test tokens were presented during training, although with lower frequency than the intended training items). We have fixed this bug and re-estimated all models. We now show model word error rates on trained and untrained tokens for direct comparison. While word error rate on the updated, truly held-out test set is generally somewhat lower than in our previous report, the new pattern further highlights the benefit of the modified loss function we introduced.

The updated Figure 4 (D and F), reflecting truly held-out test items confirms that only trained-on tokens exhibit the too-early identification. We now interpret that result as suggesting that the model learns token-specific acoustic features. Notably, the too-early activation effect is also much less pronounced for synthetic talkers (which were used in the original EARSHOT paper), suggesting that this problem is specific to the distinctiveness of human talker tokens.

In order to allow a more thorough assessment of the issue you raised, we also included a theoretical prediction of the target choice measure we plot for the models, based on phonetic transcriptions from the TextGrids in the MALD dataset (Figure 4, panels B and E).

- The presentation of the results in terms of activation values and probabilities is useful and potentially helpful. However, I still don't understand what is shown in Figure 4B, and similarly in the right hand plots of 4C, 4D, 4E, 4F). The values shown - ranging from 0 to 1 with multiple high values in the graph - do not correspond to what is described (values are individual word activation values divided by total activation and hence interpretable as probabilities which should sum to 1). I'm also confused by the fact that the target word is not included in these plots. If so, this is further reason to think that these can't be described as probabilities - the target word should be quite clearly the most probable candidate by word offset. Some changes or clarification would be very helpful.

We apologize for the lack of clarity. To address this, we have improved the descriptions of the quantities that are shown in the Figure legend to Figure 4-A and B. It now reads:

“Left panel: Activation of cohort competitors in response to human speaker words is limited (shown for the 1 layer localist model). The plot shows model output values in the localist space, interpreted as lexical “activation”, as a function of time since word onset. Shown is the output for the target (yellow), and the average for non-target words, with color indicating how many word-initial phonemes the respective non-target shares with the target. Only data for the human speaker tokens are shown (including 2751 trained and 183 untrained tokens). Values range from 0 to 1 due to the sigmoid activation function. Right panel: The relative activation of the correct target, as a function of how many word-initial phonemes the target shares with at least one other word (indicated by color). Relative activation is calculated as activation of the target, divided by the sum of the total activation in the output space, at each time point. Because the activation of the target is divided by the total activation, this can be read as a probability estimate for the target. Words that share more phonemes with competitors should take longer to reach high probability.“

> - Furthermore, I see that the x-axis on the time-course plots in Figure 4 are given in milliseconds - this is useful since it (presumably) permits alignment with Figure 2E which similarly shows the time-course of speech presentation in milliseconds. I wonder if more could be done, though, to depict the nature of the target word and/or competitors - e.g. marking uniqueness points and phoneme onsets, or aligning identification timecourses to unique points. I do think it would be helpful for readers to more fully understand the difference between an appropriate and inappropriate time-courss of activation for simulations with the conventional and modified loss functions.

This should be addressed by the simulation we have added, which shows expected target choice probability based on purely phonetic perception (Figure 4, panels B and E).

> - In Figure 4G and 4H the black line shows word error rate and neural prediction for models without the additional divisor. This is labelled as "constant", but is perhaps more accurately labelled as c=1 or or using the "original" loss function.

We have relabeled it as “c = 1 (original)”

> - Finally, statements in the results are made with regard to improved word recognition and neural prediction with the modified loss function. However, reporting of the statistics is rather sparse. I wonder if there's some way of annotating, or marking those datapoints in Figure 4G and 4H that show significantly improved performance compared to the original loss function.

We have added error bars to all word error rate plots (Figures 2, 3 and 4). In order to calculate error bars, we treated each speaker as a subject, i.e., we test whether the word error rate differences are reliable across the different speakers. We also added additional statistical tests to the main text.

Minor comments:

- In Figure 2C and the main text there's an inconsistency in whether "Sparse Random Vectors" are abbreviated SRV or RSV.

Fixed.

- Figure 3D caption "because the predictive power"

Fixed.

- Page 10 line 4-5 "the pattern in response to synthetic speakers..."

Fixed.

- They have correctly described reference [48] as showing influences of subtle acoustic cues to word length. This conference paper is largely superseded by a longer journal paper (https://doi.org/10.1037/0096-1523.28.1.218) which reports these and other related results and might be more accessible for readers.

We have replaced the reference.

- In my original review, however, I referred to a different 1997 computational modelling paper (showing activation proportional to conditional probabilities in a localist RNN trained to recognise words in speech-like sequences):

Davis, M.H., Gaskell, M. G. and Marslen-Wilson, W. D. (1997) Recognising embedded words in connected speech: Context and competition in Bullinaria, Glasspool & Houghton (Eds) Proceedings 4th Neural Computation & Psychology Workshop: Connectionist Representations. London: Springer-Verlag. https://link.springer.com/chapter/10.1007/978-1-4471-1546-5_20.

I'm happy to share a PDF if this is unavailable to the authors. They're not obliged to cite this other paper. However, they might find it informative to consider the activation time-courses shown in this paper - and their clear link with activations corresponding with conditional probability - when thinking about my comments on Figure 4 above.

Thank you for the clarification. We have added a comment to further contextualize the pattern of early target activation seen in Figure 4-A: “This early preference for targets is also inconsistent with earlier work (37) using Elman networks (6) trained to activate word outputs (with a small lexicon) from abstract phonetic feature inputs presented phoneme-by-phoneme. This network generated activations that closely matched the conditional probabilities of words given the unfolding phoneme sequence”

Signed: Matt Davis, Cambridge, UK
